# Gold Inlays

**Published:** 1907-06-15

**Authors:** T. P. Hinman


### Gold Inlays.

BY T. P. HINMAN, D. D. S.

In preparing cavities for gold inlays, the walls of molars and bicuspids should flare from the center and be well extended bucco-lingually, and the anchorages made on the occlusal surfaces only. Cut the floor wall flat with square ended fissure or inverted cone burs, leaving all walls except the cervical and retention walls in the occlusal surface converging so that the matrix can be removed without distortion. Have no lateral undercuts, and trim all margins with a sharp chisel, so that definite angles will be produced at right angles to the tooth surfaces. The margins, however, should not be made so sharp as to cut the matrix in adaptation. Use pure gold plate, two one-thousandths thick for small cavities, and three one-thousandths thick for large cavities. The plate should be rolled smooth and without wrinkles. It should always be annealed just before using. Adapt to a moist cavity with wet pledgets of cotton, which may be forced by a plugger or orange wood sticks, or an automatic plugger to place. When approximately adapted, remove the cotton and go over it with an automatic plugger directly on the gold and finish with a burnisher. Mend tears with pellets of foil or scraps of plate forced into the cracks of the matrix by pluggers while the matrix is still in the cavity. Be careful not to over burnish as you make an imperfect adaptation of the matrix. Begin filling the matrix by using 22 karat solder, and complete it with 20 karat. Remove the matrix from the cavity by filling it over full with warm modeling compound, and before removal have the patient close his teeth onto it to get the occlusion ot opposing teeth. Chill modeling compound and remove, bringing away the matrix with the compound. Invest in "sump," or other good investment material, and it is ready to fire and fill with solder. Trim to tooth form and polish, always leaving a slight fullness at the margins that may be burnished to a tight joint if the inlay should not quite fit. If not needed, it can easily be removed by strip after the inlay is set.

I set my inlays with Ames' Hydraulic Cement. In badly decayed posterior teeth where it will not show, I sometimes use Ames' Oxyphosphate of Copper. Hollow inlays are unsatisfactory, and I don't make them. Don't use too small a blow-pipe flame, for fear of burning the gold

Know exactly the thickness of your matrix material

Don't fill small cavities with gold inlays; it is a waste of time.

Be careful and don't use an excess of borax.

Don't let the solder run through onto the cavity side of the matrix.

Don't use low grade of solders, even in repair work.

Don't over contour.

Don't expect the impossible from inlays.

DISCUSSION.

DR. L. E. CUSTER, DAYTON, OHIO: While it would seem that the gold inlay has followed in the wake of the porcelain inlay, yet I know of one man who has been pounding away at the gold inlay for just as long a time. Dr. W. V. B. Ames has been harping upon the good qualities of the gold inlay for lo these many years. The technique of the gold inlay is so entirely different from that of the gold filling that few attempted the gold inlay until, having become skilled in the porcelain technic, and learning the weak points of porcelain, they took up the gold inlay. We have been using porcelain long enough, now that we can see its shortcomings as well as its good qualities. The inlay has by this time been sufficiently tested to find its place in dental practice. We have learned the principles of cavity preparation for the inlay, we have found out that porcelain can not be subjected to stress unless it is in bulk, and that thin edges soon break off. But we have at the same time found that the inlay will save teeth, and that in large cavities it is an easier operation for the patient than the making of a gold filling. And so today, as the outgrowth ot the porcelain inlay we have the gold inlay, a part of dental practice which possesses merit of a high order.

One of the chief advantages of the gold over the porcelain inlay is the fact that the matrix, being left on the inlay, we have at the very outset a more perfect fit of the stopping, and in addition to this, the gold being malleable, may, at the time of setting, be burnished into perfect contact with the enamel margin, thus reducing the cement space to a microscopical line, so that in point of a tooth-saving agent, the gold inlay is better than the porcelain. Another point of advantage is in the edge strength of gold over porcelain, whereby even frail walls may be protected rather than cut away.

Dr. Hinman's paper deals almost entirely with the technic of gold inlays. He correctly advocates the extensive use of the chisel in the preparation of the cavity, for in so doing one is more certain of securing walls without undercut. Pure gold may here be used for the matrix, which is easier worked than platinum, yet while platinum may tear when used for a porceiain filling, these openings need not cause anxiety, for porcelain will bridge them over without running through. Just the opposite is true in the making of the metal inlay; capillary attraction draws the melted metal through the breaks in the matrix, and a perfect matrix, or one whose tears have been covered with foil, is an absolute necessity for making a gold inlay.

The doctor advocates the use of modeling compound for supporting and removing the matrix, but I much prefer the sticky wax for the purpose. It is harder, and after a little heating easily drops out of the matrix. This can not be said of modeling compound. For the investment he recommends Sump. I have for years used finely ground fire clay and plaster with more satisfaction than anything else.

I, with the author, would not advocate the hollow inlay.

I object to it because it may be ground through when articulating; besides that I would object to its use on the ground that its walls, unless thick enough to make the filling practically solid, will admit of spring, and in time a hollow filling would break its attachment.

Inlay filling of both porcelain and gold has become an established method of dental practice, and the advantages and disadvantages of both materials are already showing themselves. We are even now where esthetic considerations are not material, where the conductivity of metal is not a bar to its use, in favor of the gold inlay by reason of its edge strength, its better natural fit in the cavity, and this still further enhanced by the malleability of the metal, which permits of a final burnishing into an actual contact.

H. M. Semans, Columbus, Ohio: Dr. Hinman's paper describing his method of gold inlay procedure is a very clear and concise one; here are many points which could be followed by many operators tending towards better and more careful work. This is a description of work by a man who has given thought to his methods, both the how and why, and he has the happy faculty of imparting them to others without superfluous words, and in a clear manner. There are a number of other ways and means ot constructing gold inlays which have merit and have given splendid results to all who have carefully followed out the right procedure in each case. I myself have made use of several methods, including the hollow-swedged, the mat gold and solder, and this one described by Dr. Hinman, with both good results and indifferent results; the latter, however, I believe, from taulty manipulation and not trom taulty method, as I teel sure that I have had good results from the three methods. At first I gave up the use of platinum for the very reason given by Dr. Hinman—the leaking of solder through tears and holes—but I think the platinum we get now is better adapted for matrix work, and there are times when I prefer not to invest the work. If I find a tear in the platinum ot large dimension, I have sometimes invested it and placed a little moldine over the tear, doing the same sometimes with the gold matrix. I am glad Dr. Hinman has exhibited some of his ways of contouring. Right here is found many a slip in proper work, from the lack of proper proximal contouring, there being but a haphazard attempt at proper restoration, or else the destroying of what little contouring there is, by trimming and polishing away. Dr. Hinman spoke, not only most important, but really vital, words when he said the operator must have at hand correct methods of cavity formation. It is a very necessary proposition that the operator should give the case at hand a most thorough analysis of how it should be attempted. Many are prone to be guided by their shaping the cavities from mere cleansing out of debris, and not from the fact that all inlay work requires a cavity preparation of its own class and style, several points of which the paper has brought out. I am glad to hear him admonish us on the lack of use of chisels. In many an office excavators and chisels are never used, in fact their use seems to be in many cases a lost art, if they ever had the art of manipulation of them

This is a paper and subject which deserves much discussion on the part of the society, as being of a rather new order of things, with a number of seemingly good methods. I believe we are away beyond the discussion of why we use gold inlays to-day, but we are ripe for the various hows, and we might still need some advice as to when we should use them.

Dr. Henry Barnes, Cleveland, Ohio: There is much in the paper to be commended. I do not altogether agree with the essayist's technique. I use platinum for the matrix. I furthermore use no flux whatever. Nor do I invest. Many suggestions for making porcelain inlays have no application to the making of gold inlays. My conclusion, after much experimenting, is that pure gold "sweated" into the matrix gives the best results. I do not care for a slight tear in the floor of the matrix. By my method of sweating, no gold need pass through to cause a misfit.

By applying portions of gold just where you need them to round out a contour, the sweating process may be relied on to cause it to flow exactly where you need it.

